# “Spitting Stones” and Broncholithiasis Caused by *Mycobacterium chimaera* in a Patient Affected by Bronchiectasis: A Rare Clinical Case

**DOI:** 10.1155/crpu/8029074

**Published:** 2025-11-24

**Authors:** Ambra Migliarini, Alessandra Iacovelli, Flavio Marco Mirabelli, Rosaria Capone, Maria Luisa Nicolardi, Daniela Bosco, Flavia Adotti, Alessandra Oliva, Paolo Palange

**Affiliations:** ^1^Department of Public Health and Infectious Diseases, Sapienza University of Rome, Rome, Italy; ^2^Respiratory and Critical Care Unit, Policlinico Umberto I Hospital, Sapienza University of Rome, Rome, Italy; ^3^Department of Radiological, Oncological, and Pathological Sciences, Sapienza University of Rome, Rome, Italy; ^4^Departement of Public Health and Infectious Diseases, Sapienza University of Rome, Rome, Italy

## Abstract

We describe the case of a 62-year-old woman with underlying bronchiectasis who referred to our respiratory outpatient clinic for hemoptysis and lithoptysis, in addition to difficulty gaining weight over years. Chest CT scan revealed small pulmonary nodules, bronchiectasis with mucus plugs, and calcifications, both endobronchial and peribronchial. Bronchial lavage fluid was positive for *Mycobacterium (M.) chimaera*. A daily macrolide-based triple antibiotic therapy, including rifampicin, ethambutol, and azithromycin, was started. Broncholithiasis associated with pulmonary nontuberculous mycobacterial infection is a rare clinical condition. To the best of our knowledge, this is the first report of broncholithiasis due to *M. chimaera* infection.

## 1. Introduction

Broncholithiasis is a rare condition, defined as the presence of calcified or ossified material, denoted as a broncholith, within the tracheobronchial tree. The first report of lithoptysis dates to 300 BC, when Aristotle described a symptom of “spitting of stones” [1]. Most of the authors agreed that endobronchial broncholiths originated from calcified peribronchial lymph nodes, which erode the bronchial wall [2, 3]. The causes of broncholithiasis are mainly sequalae from previous infection, mostly mycobacteriosis and histoplasmosis [4]. Broncholiths are also commonly seen from erosion of calcified granulomas from prior granulomatous disease [4, 5].

NTM are ubiquitous in the environment and can cause a wide range of infections, with pulmonary involvement being the most frequent, and accounting for 65%–90%of cases [6, 7]. NTM lung infection occurs mainly as nodular bronchiectatic or fibrocavitary disease [7], while reports of broncholithiasis from NTM are rare [8–10]. *M. chimaera* was first reported in 2004, identified as a novel NTM species belonging to the *Mycobacterium avium* complex (MAC) [11]. It was predominantly found to cause pulmonary infections in patients with chronic structural lung disease, such as bronchiectasis, chronic obstructive pulmonary disease, or cystic fibrosis and rarely sporadic cases of disseminated disease in immunocompromised hosts [12, 13]. *M. chimaera* infection could be more common and more virulent than expected in chronic respiratory diseases, as we presented in an Italian case report [14]. Even if pulmonary infections are much more common, there are other forms of infections. *M. chimaera* infections can follow open heart surgery, due to contaminated heater-cooler devices of heart–lung machines, where the mycobacteria was aerosolized and infected the surgical site, leading to localized infection or disseminated disease in immunocompromised patients with a high rate of mortality [12, 15].

## 2. Case Presentation

A 62-year-old woman referred to our respiratory outpatient clinic for mild hemoptysis and productive cough along with a history of sputum containing small round white stones and brown crystals over the previous 5 years. She also reported worsening of asthenia over the past few months and difficulty gaining weight over years, despite appropriate dietary intake. She was a teacher, her medical history was positive for bronchiectasis, diagnosed at 58 years of age, gastroesophageal reflux disease, and osteoporosis. She was an ex-smoker and had no history of atopy or dust inhalation, nor had bird or wild animals contact or recent trips.

She presented eupneic, with SpO_2_ of 98% in room air. Her body mass index was 16.1 kg/m^2^. Chest auscultation revealed fine crackles at the right basal area. Blood analysis showed absolute lymphocytopenia (670 × 10^3^ cell/mm^3^), with reduction of both B and T cells, normal CD4–CD8 ratio. HIV tested negative. Autoimmune panel and gamma globulins were normal. Aspergillus IgE and Quantiferon test were negative.

**Figure 1:**
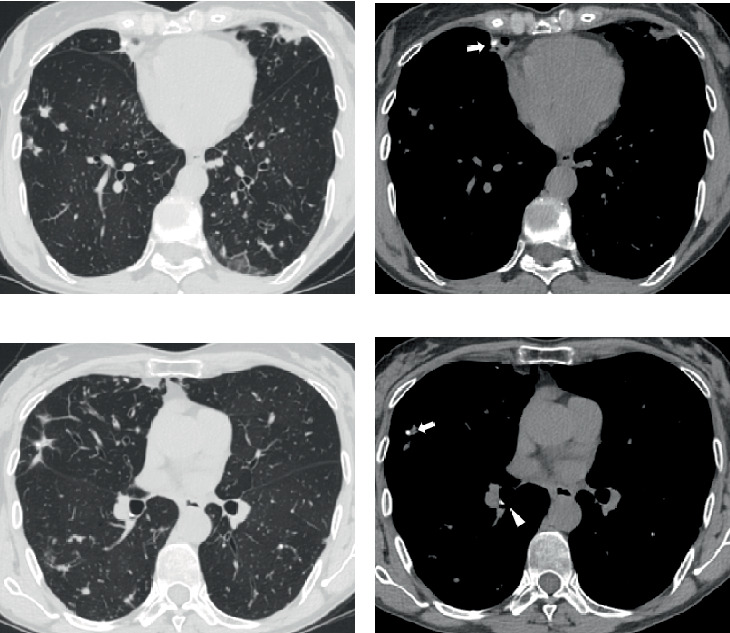
(a–d) 62-year-old woman, affected by bronchiectasis complicated by pulmonary NTM infection and broncholiths. High-resolution lung CT scan ((a, c) parenchymal window and (b, d) corresponding mediastinal window) shows multiple pulmonary nodules due to NTM infection and mild central bronchiectasis with mucus plugging (dilatation of the bronchial tree with mucoid impaction in a and c). Broncholiths are endobronchial (arrow in b and d) and peribronchial (arrowhead in d).

High-resolution computed tomography (CT) scan of the lungs showed bronchiectasis involving all pulmonary lobes, predominantly in inferior lobes and lingula, with mucus plugs and multiple pulmonary nodules, mainly in the right middle lobe, lingula, and superior segments of the lower lobes. Small calcifications, both broncholithis and peribronchial calcifications, were observed (Figure 1). Comparison with previous images obtained 5 years earlier demonstrated progression of the central bronchiectasis and pulmonary nodules and new onset of calcifications.

**Figure 2:**
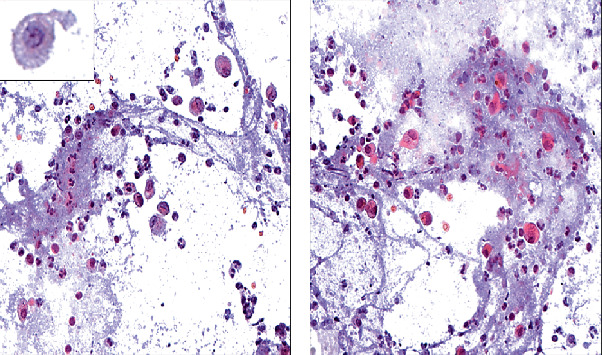
(a, b) Cytologic analysis of sample of brochoalveolar lavage fluid. Pap stain, 40x: (a) Numerous neutrophils (small round cell with segmented nucleus), foamy macrophages (insert) and rare erythrocytes (red cells). (b) Abundant necrotic debris background.

Flexible bronchoscopy was performed showing no bronchoscopic evidence of broncholithiasis likely secondary to the distal location of the calcified nodules seen on CT. Abundant secretions were present. Bronchial washings were performed. Blood samples and sputum were not collected. Cytology smears showed foamy macrophages in a “diffuse pulverulent necrotic background” (Figure 2). Microbiological culture and PCR resulted positive for *M. chimaera*. A diagnosis of broncholithiasis caused by NTM was made.

Considering the patient's symptoms and the radiological progression over time, we decided to start antibiotic therapy with rifampicin 600 mg, ethambutol 15 mg/kg, and azithromycin 500 mg, daily. She also started a pulmonary rehabilitation program and nutritional support. At 2 months of follow-up, the patient had no expectoration and reported ameliorating asthenia, even if she had difficulties in gaining weight.

## 3. Discussion

To the best of our knowledge, this is the first case of broncholithiasis caused by *M. chimaera*. No written consent has been obtained from the patients as there is no patient identifiable data included in this case report. Our patient presented with lithoptysis, a specific but rare symptom/sign of broncholithiasis, that was confirmed by chest imaging. Chest CT is essential to diagnosis of broncholithiasis, showing endobronchial calcifications, and airway obstruction such as atelectasis, mucoid impaction, or air trapping [2, 4, 5, 16]. The bronchoscopic features can be variable and range from excessive secretions to visible stones [4].

In our case, hemoptysis, fatigue, and difficulty gaining weight over years, in addition to the slow radiological progression, raised the suspicion of NTM infection. According to the NTM guidelines [17], the microbiological results of bronchial washing allowed us to make the diagnosis of infection by *M. chimaera*.

Cases of broncholithiasis associated with NTM infections are rare [4–6], outlined in Table 1. A broncholith can be the secondary effect of mycobacterial granulomatous lymphadenitis, in tuberculosis [1] and NTM disease [4], although different theories were hypothesized [5].

Management of broncholithiasis includes observation or endoscopic removal and surgery in symptomatic patients [1, 2]. The best approach remains controversial, due to possible risk of bronchial injury or massive hemoptysis, but contemporary reports describe cases successfully treated with different endoscopic techniques [1, 2, 17–19].

The decision to initiate antimicrobial therapy for NTM pulmonary infection is usually individualized, due to the difficulty of eradication and possible side effects (vision loss, renal disease, hepatic disease, ototoxicity, etc.) [20]. We started triple antibiotic therapy, macrolide-based regimen, according to the guidelines for macrolide-susceptible MAC pulmonary disease [20]. Considering the lack of evidence regarding the appropriate therapeutic strategy in this case, we decided not to adjunct parenteral aminoglycoside, as suggested for cavitary or advanced/severe bronchiectasis disease, but opted for a daily regimen, instead of three times per week.

In conclusion, pulmonary *M. chimaera* should be considered as a rare, but potential pathogen causing broncholithiasis, including in the immunocompetent host.

## Figures and Tables

**Table 1:** Case reports of broncholithiasis associated with NTM infection. F, female; M, male.

**Study**	**Age (y)**	**Sex**	**Immune status/lung disease**	**Broncholithiasis diagnosis**	**Pathogen**	**Treatment**	**Outcome**
Our study	62	F	Bronchiectasis	Lithoptysis, CT scan	*M. chimaera*	Rifampicin, ethambutol, azithromycin (daily)	Ongoing treatment
Takeda 2021 [5]	77	F	Colon cancer	CT scan, bronchoscopy	*M. intracellulare*	Bronchoscopic extraction	No recurrence
Cuppen 2007 [4]	14	M	Aplastic anemia, myelodysplastic syndrome	CT scan, bronchoscopy, autopsy	*M. kansasii, Aspergillus, Stenotrophomonas maltophilia*	Rifampicin, ethambutol, levofloxacin	Death
Martínez 1996 [6]	65	F	N/A	X-ray, bronchoscopy	*M. avium*	N/A	N/A

## Data Availability

The data that support the findings of this study are available from the corresponding author upon reasonable request.
